# New Developments in the Field of Reaction Technology: The Multiparallel Reactor HPMR 50-96

**DOI:** 10.1155/JAMMC.2005.26

**Published:** 2005

**Authors:** Arne Allwardt, Norbert Stoll, Christian Wendler, Kerstin Thurow

**Affiliations:** 1 Institut für Automatisierungstechnik Universität Rostock Richard-Wagner-Strasse 31 Rostock-Warnemünde 18119 Germany; 2 Center for Life Science Automation (CELISCA) Friedrich-Barnewitz-Strasse 4 Rostock 18119 Germany

## Abstract

Catalytic high-pressure reactions play an important role in classic bulk chemistry. The optimization of common reactions, the search for new and more effective catalysts, and the increasing use of catalytic pressure reactions in the field of drug development call for high-parallel reaction systems. A crucial task of current developments, apart from the parameters of pressure, temperature, and number of reaction chambers, is, in this respect, the systems' integration into complex laboratory automation environments.


Journal of Automated Methods & Management in Chemistry, **2005** (2005), no. 1, 26–30
© 2005 Hindawi Publishing Corporation

### New Developments in the Field of Reaction Technology: The Multiparallel Reactor HPMR 50-96

Arne Allwardt,[1] Norbert Stoll,[1] Christian Wendler,[1] and Kerstin Thurow[2]

[1] *Institut für Automatisierungstechnik, Universität Rostock, Richard-Wagner-Strasse 31, 18119 Rostock-Warnemünde, Germany;*
[2] *Center for Life Science Automation (CELISCA), Friedrich-Barnewitz-Strasse 4, 18119 Rostock, Germany*

*Received 4 June 2004; Accepted 2 August 2004*

Catalytic high-pressure reactions play an important role in classic bulk chemistry. The optimization of common reactions, the search for new and more effective catalysts, and the increasing use of catalytic pressure reactions in the field of drug development call for high-parallel reaction systems. A crucial task of current developments, apart from the parameters of pressure, temperature, and number of reaction chambers, is, in this respect, the systems' integration into complex laboratory automation environments.

### 1. INTRODUCTION

The progress in automated reaction technology is marked by the continuous development and the miniaturization of the reaction setups. The most important reasons for this development are, on the one hand, the reduction of the amount of used substances (and consequently experimentation costs), and on the other hand, the possibility of increasing the throughput, and therefore reducing the research costs.

In this respect, one also has to bear in mind that the miniaturized reaction systems often have to be modified in terms of the surface-volume ratio to fit the conventional reaction systems and to allow the possibility of a future upscale to larger reaction systems.

The decisive advantage of parallel reaction systems is the large number of reaction chambers (4-96; feasible volumes 1 to 500 mL) that allow for the simultaneous completion of several reactions.

Presently, there are various commercial systems available. Devices with a pressure level up to 10 bar are the most common. These systems generally use a shared gas supply and therefore do not allow an individual pressure setting per reactor (e.g., ASW2000 (Chemspeed), Chem-SCAN lp (HEL), Advantage Series 3400 (Argonaut)). Machines with a possible reaction pressure of more than 10 bar are usually equipped with individual pressure settings for each reaction chamber. There are, however, differences regarding the possible temperature settings (e.g., Endeavor system (Argonaut), PPR (Symyx), Chem-SCAN hp (HEL), eightfold parallel reactor (Rostock University [1, 2])).

Owing to their limited integration capabilities, the available reaction systems can only be used to some extent, if at all, in complex laboratory systems. The "high-pressure parallel reactor" by Symyx [3, 4] offers the best integration capability into existing laboratory systems due to its microplate format; however, it requires too many manual interventions with respect to a fully automated synthesis solution.

### 2. THE MULTIPARALLEL REACTOR HPMR 50-96

A robot-integrated multiparallel reaction system, originally intended for catalytic pressure reactions, was developed with the following features, listed in Table 1.

### 2.1. *Concept*

The concept described in Figure 1 is based on a modular reactor set-up, whose decentralized conception includes the shifting of control tasks to individual, functionally coherent units. Thus the structural units of the gas management system (pressure management), the temperature management, and the homogenization (mixing) possess separate control modules, whose intelligence allows an independent control and adjustment of the structural units following the transmission of the target parameters. This approach also reduces the workload of the central control server considerably. The communication between the decentralized parts of the control system is realized through serial RS232 interfaces attached to the individual structural units.

To operate the reactor, 230V/50 Hz power sockets as well as the usual laboratory surroundings of compressed air, vacuum, inert gas, and reaction gas are required.

Correspondence and reprint requests to Norbert Stoll, Institut für Automatisierungstechnik, Universität Rostock, Richard-Wagner-Strasse 31, 18119 Rostock-Warnemünde, Germany; E-mail: norbert.stoll@uni-rostock.de.

Table 1: Intended features of the high-pressure parallel reactors.

| | |
|---|---|
| Reaction chamber | Glass microtiter plate |
| Reaction volume | 96 × 200–300 $\mu$L |
| Pressure | To 50 bar absolute pressure; same pressure in each reactor |
| Temperature | 0 to 100°C; same temperature in each reactor |
| Mixing | Magnetic stirring |
| Inert conditions | Reactor filling under inert conditions<br>Preservation of inert conditions until reaction start |
| Operation modes | Stand-alone and robot integrated |

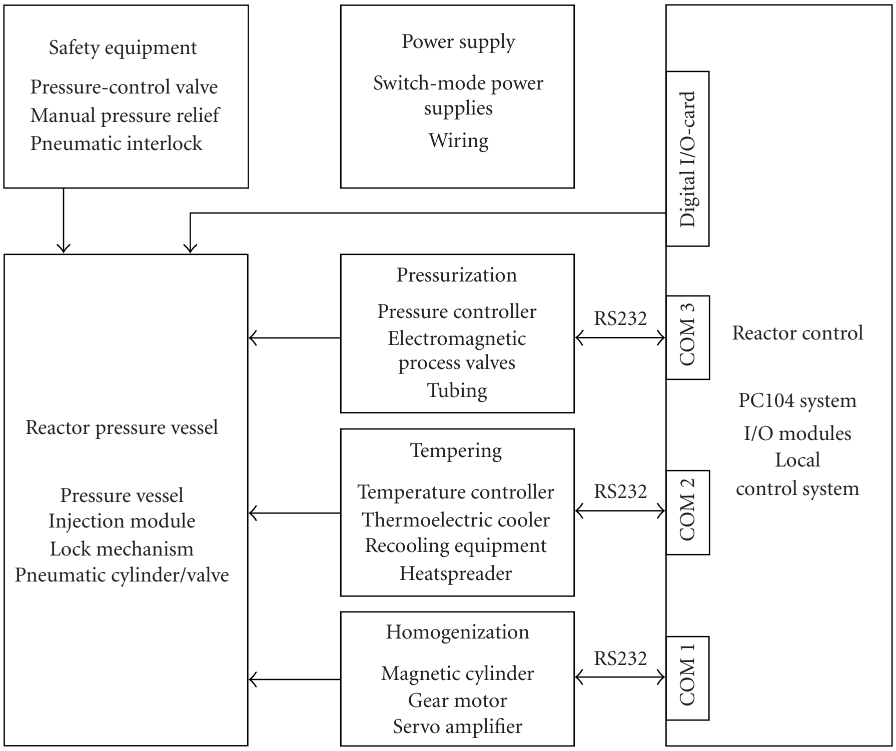

Figure 1: Conceptual structure of the high-pressure parallel reactors.

### 2.2. *Reaction chamber*

A glass microtiter plate (Zinsser Analytik) is used as a reaction chamber, which is notable because of its high thermal stability and high resistance against many chemicals. The outer dimensions of the glass reactor plates are in accordance with the SBS standards [5]. The plate used has 96 cavities, each with a volume of 530 $\mu$L and a height/width ratio of approximately 3/2, which is useful for successful upscaling.

The reaction chamber must be air- and watertight to avoid cross-contamination. This is achieved through the use of a sealing material (Santropene). A 1 mm thick stainless steel clamp allows the attachment of the ribbed mat on top of the microtiter plate, even despite the buildup of high vapor pressure in the wells.

### 2.3. *Pressure chamber*

The pressure chamber, which is made of Inconel 625 [6, 7, 8], represents the central structural unit of the reactor system, where the microtiter plate can be placed for the reaction (Figure 2). The desired robotic integration of the system is guaranteed by the bifurcation of the chamber into a movable lid and a static bottom half. The sealing of the lid onto the chamber bottom is achievable using an O-ring, which remains tight due both to the pressure between the lid and the bottom half as well as to additional deformation from inner pressure [9].

After the chamber is closed, both units are locked together with pneumatically moving arms. An injection module fastened to the inner side of the lid is used to assist in controlling the system's inert gas conditions.

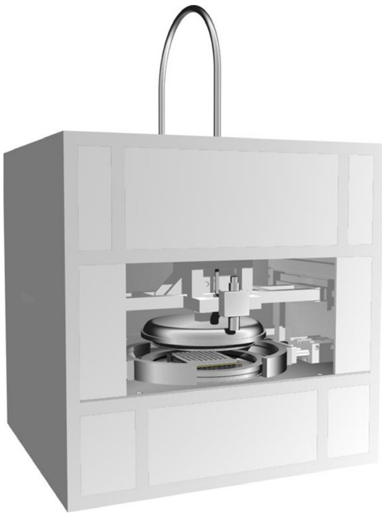

Figure 2: HPMR 50-96.

### 2.4. Homogenization and temperature

The homogenization of the reaction mixture is achieved through the use of a mixing system based on an outer magnetic field. The speed of the stirring can be as high as 500 U/min [10]. Many diverse publications have confirmed the efficiency of this principle [11].

The temperature of the reaction chamber can be varied within the range of 0°C to 100°C using six peltier elements. The heat energy that has been extracted from the chamber is released through an integrated back cooling system into the surroundings. Two specially formed copper plates assist the enlargement of the area for the heat transfer.

### 2.5. Gas management

The use of reaction gas (hydrogen or carbon monoxide) requires an effective and reliable gas management system (Figure 3). The system must ensure the inert conditions up to the pressurization of the reaction gas as well as its safe handling. The reaction gas pressure can reach as high as 50 bar within the integrated gas management system. The use of a digital pressure control device with an adaptable regulation valve allows a constant pressure rise in the chamber while avoiding pressure eruptions. Moreover, assurance of the system's performance requires a guaranteed vacuum supply. The different gas pressures can be separately controlled.

### 2.6. Control system hardware

For the central reactor control system, which both communicates with all of the other structural units of the decentralized system and supplies their sensors and actuators, a PC104 system is used [12]. With the help of the installed software, the system supports the stand-alone as well as the robot-integrated mode.

The central module is a 3.5 inch PC-board "PCM-5825" (Advantech). An integrated digital I/O card PCM-3730 uses the 16 optically isolated inputs and outputs to control the pneumatics valves as well as to read the signals of the position encoder. Two additional ORS-cards (BMC) increase the number of I/O ports for additional control options.

### 3. PROCESS CONTROL SYSTEM SOFTWARE

The flexibility of the software in relation to its enlargement and especially its ability to be transferred into future device platforms, along with the integration of the required system modes, play an important role in the conception of the reactor control system. The control system, which is divided into three levels, meets these requirements due to its modular structure and its clear organization in response to concrete assignments. Control of the experiment process is based on a method produced beforehand by the guiding system. The operation of the reactor in a higher-ranking system is achieved through the selection and initiation of a method from a chosen group of methods.

A graphical interface is used for data input and visualization. In addition to displaying the tests of the current experiment along with the parameters, the software is also capable of monitoring the reaction; it shows the current pressure, temperature, and stirring speed rates. In addition, the progress of each experiment can be observed. Moreover, a visualization of the communication between the process control system and the master system is also possible. The method planning is done through the creation of the method itself with the help of the Editor, depicted in Figure 4.

This part of the process consists of the selection of the required control commands and the arrangement of its parameters within the necessary time constraints. Every method produced is saved in a method directory in the system.

### 4. APPLICATION

The system HPMR 50-96 has been tested for the hydrogenation of benzoin to hydrobenzoin carried out by using the homogenous catalyst 1,1′-Bis(di-i-propylphosphino) ferrocene (1,5-cyclooctadiene)rhodium(I) tetrafluoroborate $[(COD)Rh(DiPFc)]^{+}BF_4^{-}$ in methanol (Figure 5).

To measure the precision under identical conditions, all 96 wells of the microtiter plate were filled with an identical solution (catalyst concentration 0.4 mol%), and the reaction was carried out at 25°C, 30 bar, and at a stirring speed of 300 U/min for 60 minutes. GC/MS analysis was used to track the reaction.

In the results, an average yield of Hydrobenzoin between 89% and 92% was detected. The precision was determined to be very high with relative standard deviations lower than 2%. In addition, it was demonstrated that repeated experiments (> 10 plates) showed very good results. Relative standard deviations of approximately 2% were measured for all plates with a yield of about 90% of R,R-, S,S-, and meso-hydrobenzoin.

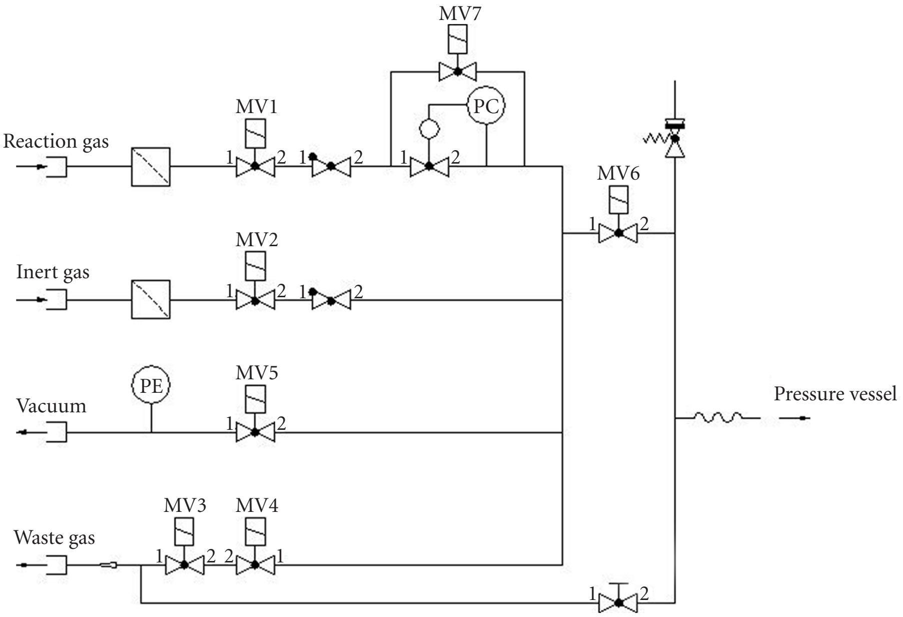

Figure 3: The concept of the gas management system.

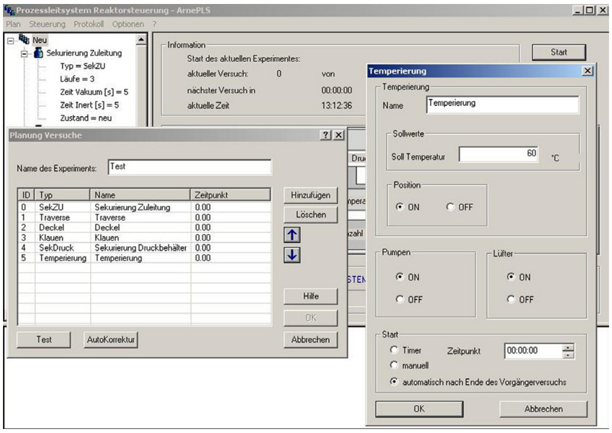

Figure 4: Editor of method planning.

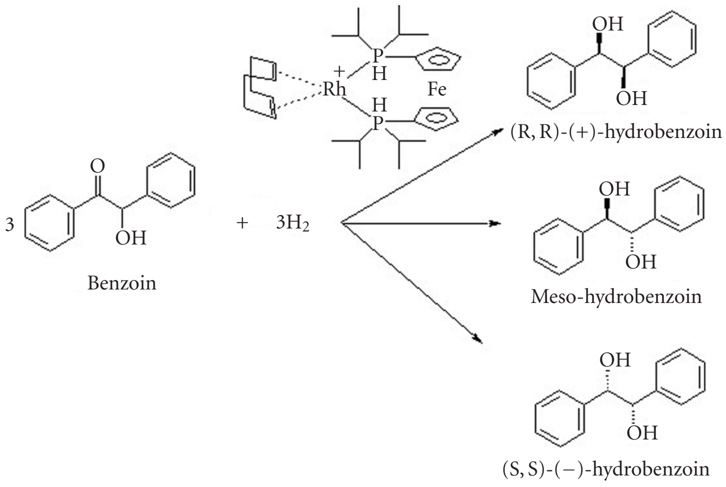

Figure 5: Formal scheme of the hydrogenation of benzoin to hydrobenzoin.

### 5. SUMMARY AND OUTLOOK

The multiparallel reactor HPMR 50-96 represents a significant development in the field of reaction technology for chemical applications. Due to its structural set-up, its modular structure, and the design of the control software, it can be used both in stand-alone mode and as an integrated component of more complex laboratory automation systems, supported by robots. The potential applications of the reactor are not limited to catalytic high pressure reactions; the system can also be used for different tasks in the field of synthesis optimization or combinatorial chemistry.

Future research projects include the expansion of the system to allow parallel treatments of up to 384 tests and the increase of the possible pressure ranges.

### REFERENCES

[1] B. E. Bosch, T. Riermeier, U. Dingerdissen, et al., "Array of autoclaves," patent DE 100 49 078 A1 und WO 2002030557 A2, 2000.
[2] A. Allwardt and N. Stoll, "New developments in microreactor technology for life science application," in *Proc. Internationaler Ausstellungskongreß für Chemische Technik, Umweltschutz und Biotechnologie (ACHEMA 2003)*, p. 32, Frankfurt, Germany, May 2003.
[3] Symyx Technologies Inc., "High pressure parallel reactor," US patent 619416, 2000.
[4] Symyx Technologies Inc., "High pressure parallel reactor," EP patent 1174185 A3, 2001.
[5] T. Astle, "Microplate standardization report," *Journal of Biomolecular Screening*, vol. 3, no. 1, pp. 3–8, 1998.
[6] N. Boukis, M. Schacht, and E. Dinjus, "Werkstoffkorrosion in überkritischen wässrigen Lösungen," *Nachrichten Forschungszentrum Karlsruhe*, vol. 1, pp. 81–90, 2001.
[7] Verband der Technischen Überwachungs-Vereine e. V., "Druckschrift: Allgemeine grundsätze für werkstoffe," Admerkblatt w0, 2002.
[8] U. Heubner and M. Köhler, "Hochlegierte Werkstoffe für besondere Beanspruchungen," VDM Report 26, Thyssen-Krupp VDM, Werdohl, 2002.
[9] B. Thier and W. H. Faragallah, *Handbuch Dichtungen*, Faragallah Verlag, Sulzbach, Germany, 1990.
[10] V&P Scientific Inc., "Magnetic tumble stirring methods, devices and machines for mixing in vessels," US patent 6176609 B1, 2001.
[11] P. Cleveland, "Microplate stirring technology," *American Laboratory News Edition*, vol. 30, no. 7, p. 10, 1998.
[12] H. J. Blank, *Das Embedded PC Handbuch*, Franzis Verlag GmbH, Poing, Germany, 2000.

